# A 43-kDa TDP-43 Species Is Present in Aggregates Associated with Frontotemporal Lobar Degeneration

**DOI:** 10.1371/journal.pone.0062301

**Published:** 2013-05-21

**Authors:** Patrick J. Bosque, Philip J. Boyer, Priya Mishra

**Affiliations:** 1 Division of Neurology, Denver Health Medical Center, Denver, Colorado, United States of America; 2 The Eleanor Roosevelt Institute, University of Denver, Denver, Colorado, United States of America; 3 Department of Neurology, University of Colorado School of Medicine, Aurora, Colorado, United States of America; 4 Department of Neuropathology, University of Colorado School of Medicine, Aurora, Colorado, United States of America; UMCG, Netherlands

## Abstract

The transactive response DNA-binding protein (TDP-43) is a major component of the abnormal intracellular inclusions that occur in two common neurodegenerative diseases of humans: (1) a subtype of frontotemporal lobar degeneration and (2) amyotrophic lateral sclerosis. Genetics, experiments in cultured cells and animals, and analogy with other neurodegenerative diseases indicate that the process of TDP-43 aggregation is fundamental to the pathogenesis of these 2 diseases, but the process by which this aggregation occurs is not understood. Biochemical fractionation has revealed truncated, phosphorylated and ubiquitinated forms of TDP-43 in a detergent-insoluble fraction from diseased CNS tissue, while these forms are absent from controls. However, a large amount of the normally predominant 43-kDa form of TDP-43 is present in the detergent-insoluble fraction even from control brains, so it has not been possible to determine if this form of TDP-43 is part of pathological aggregates in frontotemporal lobe degeneration. We used semi-denaturing detergent-agarose gel electrophoresis to isolate high molecular weight aggregates containing TDP-43 that are present in the cerebral cortex of individuals with frontotemporal lobar degeneration but not that of controls. These aggregates include the same covalently modified forms of TDP-43 seen in detergent-insoluble extracts. In addition, aggregates include a 43-kDa TDP-43 species. This aggregated 43-kDa form of TDP-43 is absent or present only at low levels in controls. The presence of 43-kDa TDP-43 in aggregates raises the possibility that covalent modification is not a primary step in the pathogenic aggregation of TDP-43 associated with frontotemporal lobar degeneration and amyotrophic lateral sclerosis.

## Introduction

Frontotemporal lobar degeneration (FTLD) is a dementia syndrome defined by both clinical and pathological features. The prevalence of FTLD is approximately 15/100,000 persons aged 45–65 years [1]. In greater than 95% of FTLD cases, abnormal intracellular protein aggregates are found in the brain [2], [3], but the protein that forms the major component of the aggregate differs between sub-types of FTLD. In 33–61% of FTLD cases, the microtubule-binding protein tau predominates in inclusions [2], [4], [5], [6]. Most of the remaining 39–77% of FTLD cases had been classified as “FTLD with ubiquitinated inclusions” (FTLD-U) because the protein aggregates were identified by the presence of ubiquitin, a small protein that covalently binds to proteins targeted for degradation [7], [8]. Recently, TDP-43 has been found to be a major component of the ubiquitinated inclusions in about 90% of FTLD-U cases [6], [9]. These cases are now denoted “FTLD-TDP.” In most of the remaining FTLD-U cases, “fused in sarcoma” (FUS), a protein with functional similarity to TDP-43, is a major component of the inclusions [4], [10], [11], [12].

Remarkably, abnormal intracellular accumulations of TDP-43 are also prominent in most cases of amyotrophic lateral sclerosis (ALS), a condition in which motor neurons of both the brain and the spinal cord degenerate, causing progressive weakness of skeletal muscle [9]. There is clinical overlap between FTLD and ALS, and TDP-43 aggregates are found in the CNS of patients who show signs of both FTLD and ALS. Mutations in the *TARDBP* gene, which encodes TDP-43, are linked to rare familial forms of ALS [13], [14], [15]. This finding strongly implicates abnormal TDP-43 function or processing in the pathogenesis of both ALS and FTLD-TDP. TDP-43 normally plays a role in RNA processing [16]. Whether FTLD-TDP and ALS are caused by loss of normal TDP-43 function or a gain of toxic activity is not established.

Serial extraction of brain homogenate in buffers of increasing stringency yields a fraction that is insoluble in a solution of 500 mM NaCl and 1% N-lauroylsarcosine [9], [17]. From FTLD-TDP brains, this detergent-insoluble fraction is enriched in covalently modified forms of TDP-43 [9]. These TDP-43 species include a phosphorylated 45-kDa form; several truncated and phosphorylated carboxy-terminal forms of approximately 25-kDa and 37-kDa; and higher molecular weight ubiquitinated and phosphorylated forms that migrate as a broad band in sodium dodecyl-sulfate polyacrylamide gel electrophoresis (SDS-PAGE) gels. The same detergent-insoluble fraction from control brains lacks forms of TDP-43 with obvious covalent modifications, suggesting that the pathological aggregates identified histologically in FTLD-TDP brains contain the phosphorylated, ubiquintinated and truncated forms of TDP-43 seen in the detergent-insoluble fraction. Immunohistochemical studies of FTLD-TDP brains confirm that intracellular aggregates contain phosphorylated forms of TDP-43 [18].

Problematically, the detergent-insoluble fraction from both FTLD-TDP and control brains harbors a large amount of 43-kDa TDP-43 [9]. It is therefore not certain whether this normally predominant, full-length form of TDP-43 is a component of pathological aggregates in FTLD-TDP. This question is important, because the TDP-43 species that comprise the aggregates may reflect the still poorly understood pathological processes that lead to TDP-43 aggregation. Specifically, phosphorylated or truncated TDP-43 species may be particularly aggregation prone. It has therefore been suggested that aberrant phosphorylation or truncation of TDP-43 may be a proximal cause of TDP-43 aggregation and subsequent neurodegeneration in FTLD-TDP and ALS [18], [19], [20], [21], [22]. The presence of 43-kDa TDP-43 in aggregates would support a hypothesis that the observed covalent modifications of TDP-43 are not the primary cause of aggregation, but might rather be cellular responses to the presence of aggregates of the full length form of the protein, initially lacking covalent modification.

To better characterize the TDP-43 species in FTLD-associated aggregates, we employed an alternative method to detergent extraction, semi-denaturing detergent agarose gel electrophoresis (SDD-AGE), to isolate high-molecular-weight complexes containing TDP-43. We then analyzed the components of these complexes by SDS-PAGE and immunoblotting. In addition to covalently modified forms, described above, we found elevated levels of a 43-kDa TDP-43 species in the FTLD-TDP aggregates.

## Results

### SDD-AGE immunoblots demonstrate non-covalently linked aggregates containing TDP-43

**Figure 1:**
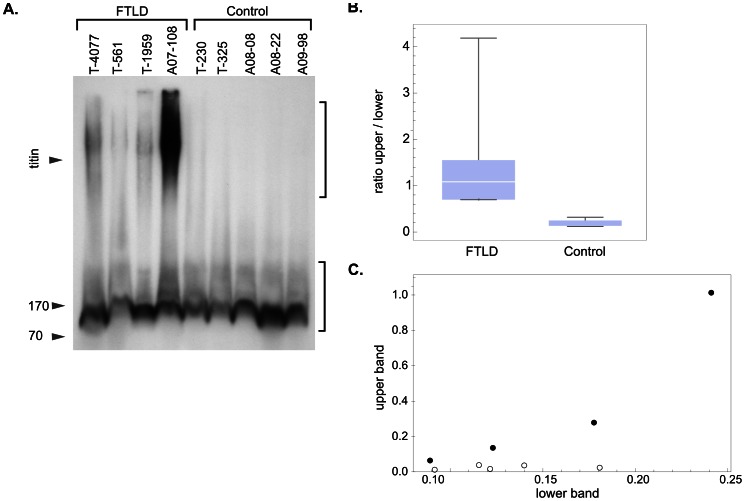
High molecular weight TDP-43 band on SDD-AGE gels found in FTLD-TDP but not control brain homogenates. (A) Immunoblot of brain homogenates separated on an analytic SDD-AGE gel and probed with polyclonal anti-TDP-43 serum. Twenty-five µL of a 10% brain homogenate from the frontal or anterior temporal cortex was loaded in each lane. A slow-migrating band (upper bracket) is present only in the FTLD-TDP cases. Arrowheads indicate the position of molecular weight markers. The arrowhead labeled “titan” marks the position of the ∼ 3000-kDa titan band on a Coomassie-stained gel of chicken muscle extract on an SDD-AGE gel run under identical conditions. (B) Ratio of upper to lower band greater in FTLD than in controls. The quantities of TDP-43 in the range of the upper bracket and the lower bracket (Fig. 1A) were determined as described in the Methods, and then the ratio of the upper to the lower band calculated. This ratio is greater in every FTLD case than in any control and the difference between the two groups is significant (P = 0.013, one-way t-test). The plots depict the range (thin horizontal bars), median (white line) and 25–75 percentile range (box) of values. (C) TDP-43 in upper band correlates with FTLD diagnosis, not with total quantity of TDP in sample. Quantity of TDP-43 in the lower band plotted against that in the upper band. Filled circles are FTLD cases, open circles are control cases. While there appears to be a correlation of TDP-43 quantity in the upper region with that in the lower for FTLD cases, no such correlation exists for controls. Quantities in the lower region for FTLD cases and controls largely overlap.

SDD-AGE has been described as a means of revealing the aggregated proteins known as “yeast prions” [23], [24], [25]. Yeast prions may bear important structural similarities to protein aggregates in human neurodegenerative diseases. We therefore examined whether SDD-AGE could be used to separate the aggregated forms of TDP-43 found in FTLD from the forms found normally in CNS tissue. The SDD-AGE procedure exposes tissue homogenates to the moderately denaturing condition of a 1% solution of the ionic detergent sodium dodecyl sulfate (SDS) and 12.5 mM dithiothreitol (DTT) at room temperature. This is followed by agarose gel electrophoresis, which resolves large aggregates from smaller species. We examined the migration of TDP-43 in SDD-AGE, comparing homogenates of anterior frontal or temporal lobe cerebral cortex from patients with histopathologically diagnosed FTLD-TDP to cortex from the same regions of control brains without histopathological evidence of neurodegenerative disease. Immunoblots of these SDD-AGE gels, probed with a polyclonal antiserum recognizing TDP-43, showed a broad band migrating at an apparent modal molecular weight of >3000-kDa in 4 FTLD-TDP cases but not in 5 controls (Fig. 1A).

In some control cases a “tail” of TDP-43 signal extended into the upper regions of the gel. To objectively confirm the difference in TDP-43 migration between cases and controls, we compared the ratio of the relative quantity of TDP-43 in a segment extending from 5 mm to 30 mm below the well (encompassing the densest portion of the broad high molecular weight band), to the relative quantity in a 20 mm segment encompassing the TDP-43 signal in the lower portion of the lane that was present in all samples. This ratio was greater in every FTLD-TDP case than in any control (Fig. 1B), and the difference between the two groups was significant (P = 0.013, one way T-test). Similarly, we investigated whether the high molecular weight band seen in FTLD cases might be the product of (hypothetically) greater amounts of TDP-43 in FTLD homogenates relative to controls. Although the total amount of TDP-43 varied between samples, the range of amounts of TDP-43 in the lower region was similar between FTLD-TDP and control groups (Fig. 1C). The amount of TDP-43 in the upper region correlated with that in the lower region among FTLD-TDP cases, but not controls (Fig. 1C). The high molecular weight band is thus specific to FTLD cases.

**Figure 2:**
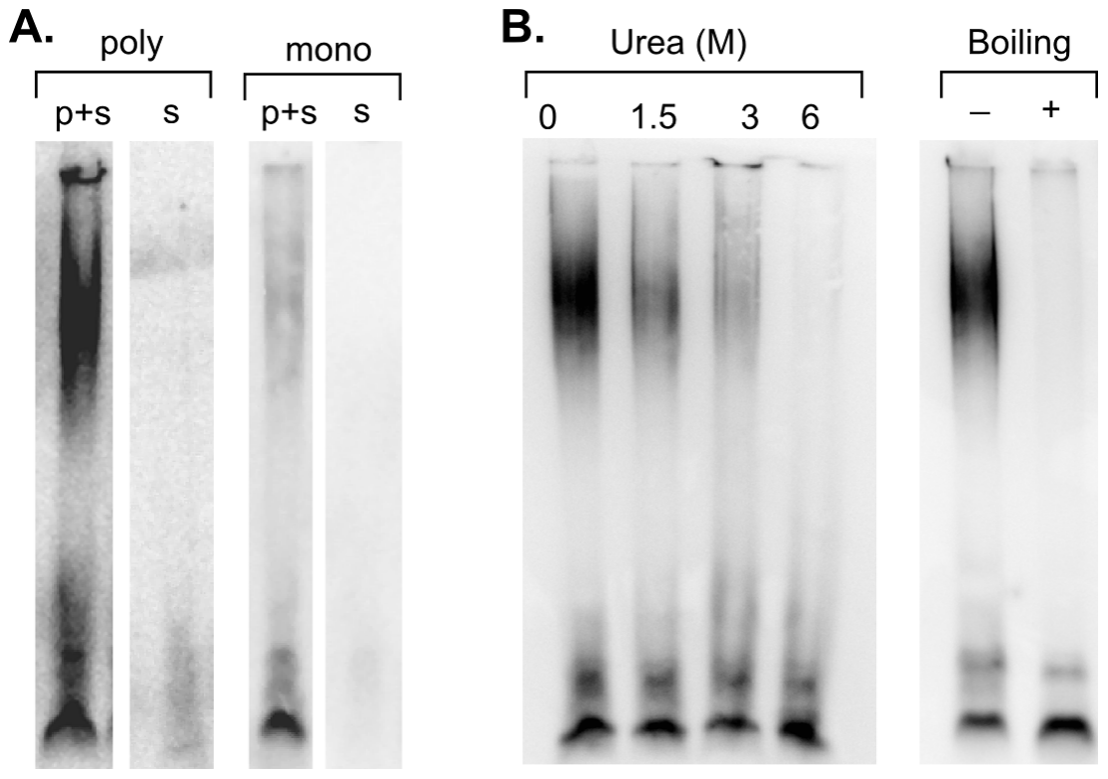
TDP-43 is non-covalently bound in a high molecular weight aggregate in FTLD-TDP. (A) Independently derived antibodies to TDP-43 bind to high-molecular weight band. Immunoblots of 25 µL of brain homogenate from a case of FTLD-TDP, probed with two distinct primary and secondary antibody combinations (p+s), or the secondary antibody alone (s): either polyclonal (poly) anti-TDP-43 serum and an HRP-anti-rabbit IgG conjugate, or a murine monoclonal antibody (mono) directed against TDP-43 and HRP-anti-mouse IgG. Both primary antibodies recognize the same high molecular weight band, while neither secondary antibody gives significant signal in this region when used alone. (B) Denaturation eliminates the high molecular weight band. Immunoblots of semi-denaturing agarose gels probed with TDP-43 antiserum. For urea denaturation, homogenates were incubated in the indicated concentrations of urea for 1 hour then before loading on the gel. For the boiling study, samples were mixed with loading buffer then either incubated at room temperature for 10 minutes or heated to 100°C for 5 minutes. Boiling in 1% SDS/12.5 mM DTT or incubating with 6 M urea eliminates most of the high molecular weight TDP-43 band.

The FTLD-associated high molecular weight band was present when immunoblots were probed with either a polyclonal antiserum or an independently derived monoclonal antibody to TDP-43, but not when blots were probed with the secondary antibody alone (Fig. 2A). Similarly, pre-absorbing the polyclonal antiserum with the immunizing antigen eliminated the slow-migrating band in immunoblots (data not shown). We subjected samples to harsher denaturing treatments prior to loading them on the SDD-AGE gel. Either boiling the sample in loading buffer (1% SDS, 12.5 mM DTT, 40 mM Tris pH 6.8) for 5 minutes or incubating it for 1 hour in 6 M urea eliminated the high molecular weight band (Fig. 2B). Since urea is a chaotropic agent that would be expected to denature proteins without disrupting covalent bonds, we conclude that the broad high molecular weight band is formed of a non-covalently linked aggregate containing TDP-43, or derivatives of TDP-43, that is stable in the loading buffer at room temperature.

### Concentration of proteins from SDD-AGE gel sections

**Figure 3:**
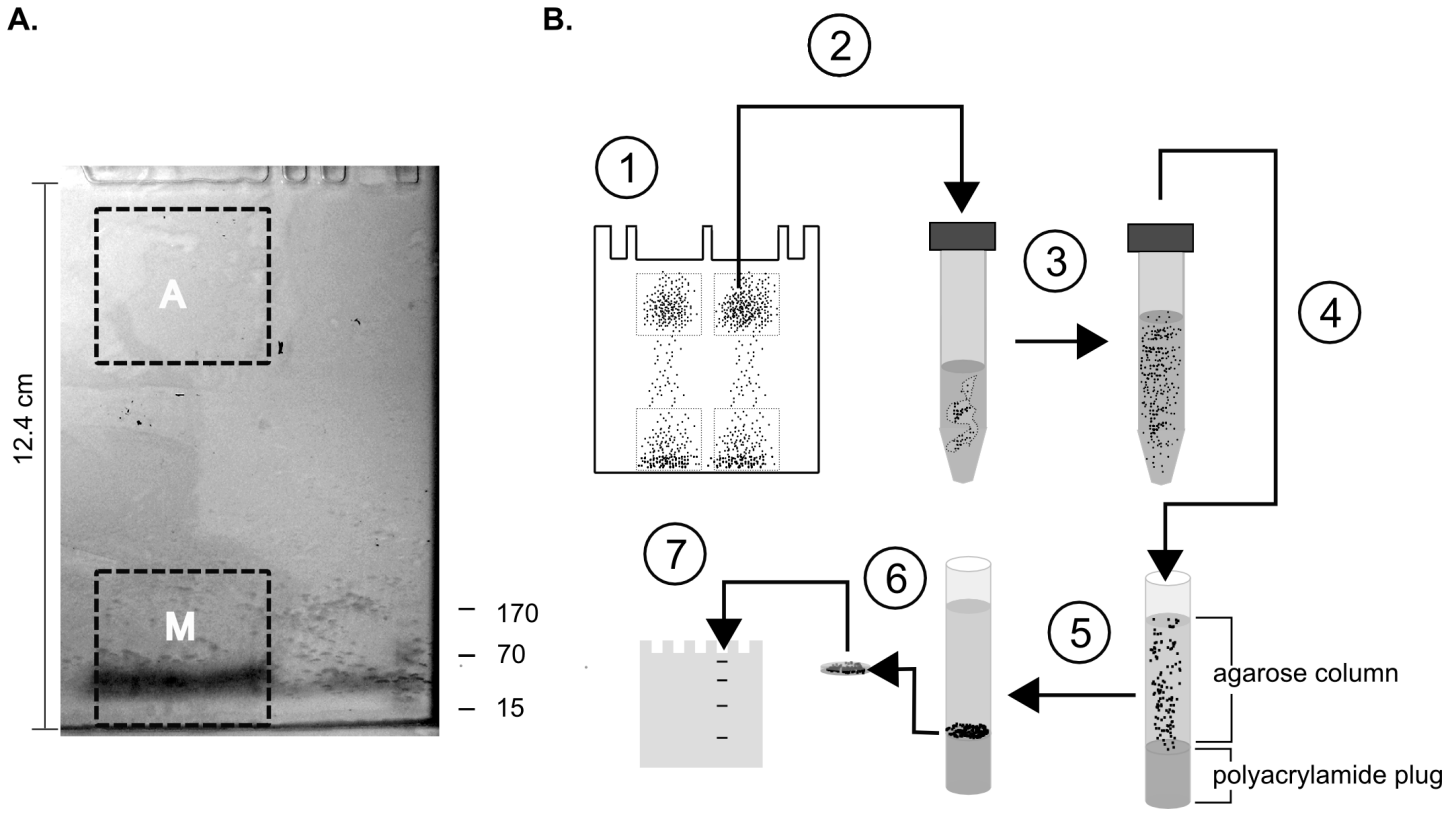
The method for analyzing components of aggregates isolated on SDD-AGE gels. (A) An unstained large format SDD-AGE gel after a typical preparative electrophoretic run. The dashed boxes depict the regions that were excised for concentration and analysis of proteins. The upper box labeled “A” circumscribes the “aggregate region” in which the major portion of aggregated TDP-43 associated with FTLD-TDP is found. The lower box labeled M contains the presumably monomeric TDP-43 forms found in both controls and FTLD cases (“monomer region”). The wide dark band at the bottom of the gel is bromphenol blue. Positions of pre-stained protein mass standards are indicated on the right. (B) Schematic depiction of the methods employed to analyze components of the TDP-43 aggregate region. The figure demonstrates the most commonly used method. Variations, when employed, are described in the text. (1) Brain homogenate (400 μg total protein) subject to electrophoresis on an SDD-AGE gel. The black dots represent TDP-43 and other proteins separated by the method. (2) The region in which the aggregate predominantly migrates is excised and transferred to a 15 mL conical tube. SDS is added to 1% and DTT to 12.5 mM. (3) Excised region of gel boiled and the volume brought to 4 mL with ultrapure water. (4) Molten agarose is transferred to glass tube with a 40% polyacrylamide plug at bottom. (5) Proteins are electrophoretically concentrated to bottom of agarose column. (6) The ∼1.5 mm (∼120 µL) section of agarose immediately adjacent to the polyacrylamide plug is excised. (7) Laemmli buffer (4×) added and the concentrated proteins are separated on a 10% polyacrylamide SDS-PAGE gel (typical volume loaded 35 µL for immunoblotting). SDS-PAGE gels further analyzed by silver staining or immunoblotting (not depicted).

We devised methods to investigate which TDP-43 species contribute to the aggregates isolated by SDD-AGE. First, we used large format gels to increase the amount of brain homogenate we could separate by SDD-AGE (Fig. 3A). We then developed a method to concentrate proteins from large excised regions of these gels into volumes small enough for analysis by SDS-PAGE (Fig. 3B). We examined the efficiency of these methods by separating 500 μg (total protein) brain homogenate on a large format SDD-AGE gel. We then excised two 3.5 cm long sections from the lane: the first region corresponding to the major portion of the high molecular weight band of TDP-43 seen in FTLD-TDP cases (which we term the “aggregate region”) and the second region comprising the bottom 3.5 cm of the lane, which includes the major TDP-43 species present in all homogenates (which for convenience we term the “monomer region”). Proteins in the excised sections were concentrated to the bottom of agarose columns as described in the Methods. Approximately 1.5 mm sequential slices were taken from the bottom of the column for analysis. We were unable to obtain reliable measures of the amount of protein in concentrates of the aggregate region by bicinchoninic acid or Coomassie assays (data not shown). Therefore, protein concentrations were estimated by comparing whole-lane densities of samples to those of standard dilutions of brain homogenates of known protein concentration on silver stained SDS-PAGE gels. The lowest 1.5 mm column slice from either the aggregate or monomer region contained approximately 85% of detectable protein from that region, with 15% in the second slice. No protein was detected in higher sequential slices.

To estimate the overall recovery efficiency of this method, we loaded pre-stained molecular weight standards (Pageruler, Fermentas) on a large format SDD-AGE gel. Unlike brain homogenates, all proteins from the pre-stained marker could be observed to run in a relatively small region toward the bottom of the SDD-AGE gel. We excised the markers in a 3.4 cm long fraction of the lane and concentrated the markers using the method described above. A sample from the final 2 mm of the concentrating column and dilutions of pre-stained standards were compared on SDS-PAGE gels. Approximately 91±11 (s.d.)% of the marker was recovered in the final 2 mm section. Since 85% of the total detectable brain protein was found in the final 1.5 mm of the column, and approximately 90% of total protein was recovered from the final 2 mm, we conclude this method efficiently concentrates approximately 77% of the total protein in an excised SDD-AGE gel region into the final 1.5 mm of the column.

### Protein composition of high molecular weight region of SDD-AGE gels

**Figure 4:**
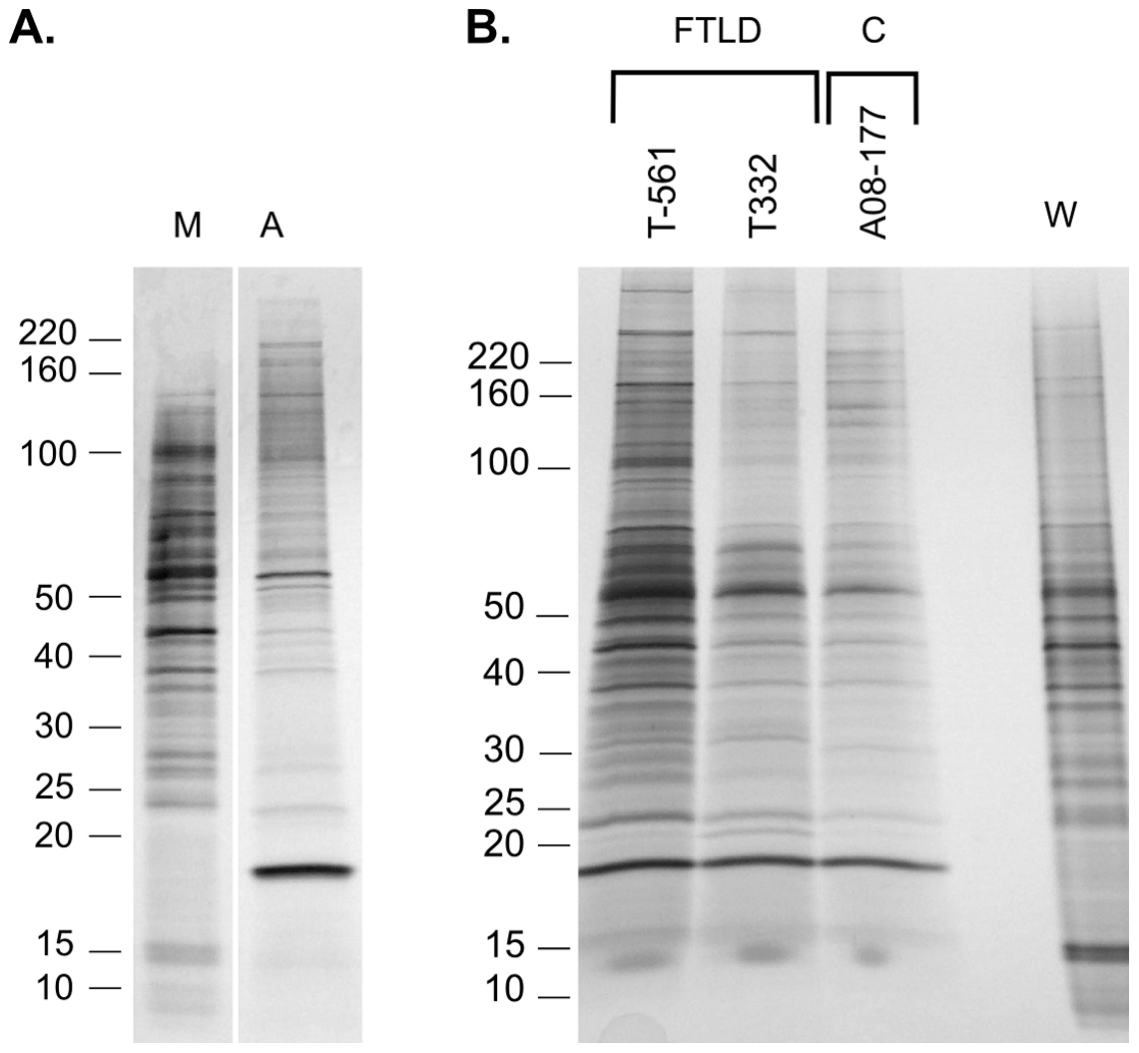
Protein components of the aggregate and monomer regions of SDD-AGE gels. (A) Proteins concentrated from aggregate or monomer regions of an FTLD case compared. Silver stained SDS-PAGE gel with equal volumes of a 40-fold dilution of the monomer region concentrate (M) and undiluted aggregate region concentrate (A) are loaded on the gel. These fractions comprise different proteins. Semi-quantitative analysis indicates the total protein content of the aggregate fraction is approximately 1% that of the monomer fraction. Bars on the left indicate the migration of molecular weight protein standards of the depicted masses (kDa). (B) Comparison of proteins concentrated from the aggregate region of SDD-AGE gels for two FTLD cases and a control (the volume loaded is ∼22% of the concentrate from a region such as in figure 3a). The lane labeled W contains approximately 1 µg (total protein) from a whole brain homogenate for comparison. The aggregate regions of either FTLD cases or controls comprise a complex mixture of proteins. No consistent difference in the band pattern is seen between cases and controls. The TDP-43 bands seen in immunoblots of proteins from the aggregate region of FTLD cases are not distinctly seen in these silver stained gels.

We compared proteins concentrated from the aggregate and monomer regions on silver stained SDS-PAGE gels. The aggregate region contains a complex mixture, with at least 40 individual protein bands revealed by silver stain (Fig. 4A). The silver stain band pattern from the aggregate region did not differ between FTLD-TDP cases and controls (Fig. 4B), however the band pattern of the aggregate region was different from that of the monomer region (Fig. 4A). A comparison of concentrates from the aggregate region to dilutions of concentrates from the monomer region on silver stained SDS-PAGE gels demonstrated that the total amount of protein in the aggregate region was ∼1% of that in the monomer region (Fig. 4A). The concentration of individual protein species in the aggregate region is also low, with only one silver stain band in that fraction being significantly darker than the darkest bands in a 40-fold dilution of the monomer fraction. Protein bands migrating in the 100−300-kDa range appear to be enriched in the aggregate fraction, suggesting that large proteins may not be completely separated from very large (>1000-kDa) proteins and protein complexes on our SDD-AGE gels.

### Aggregated forms of TDP-43 include a 43-kDa species

**Figure 5:**
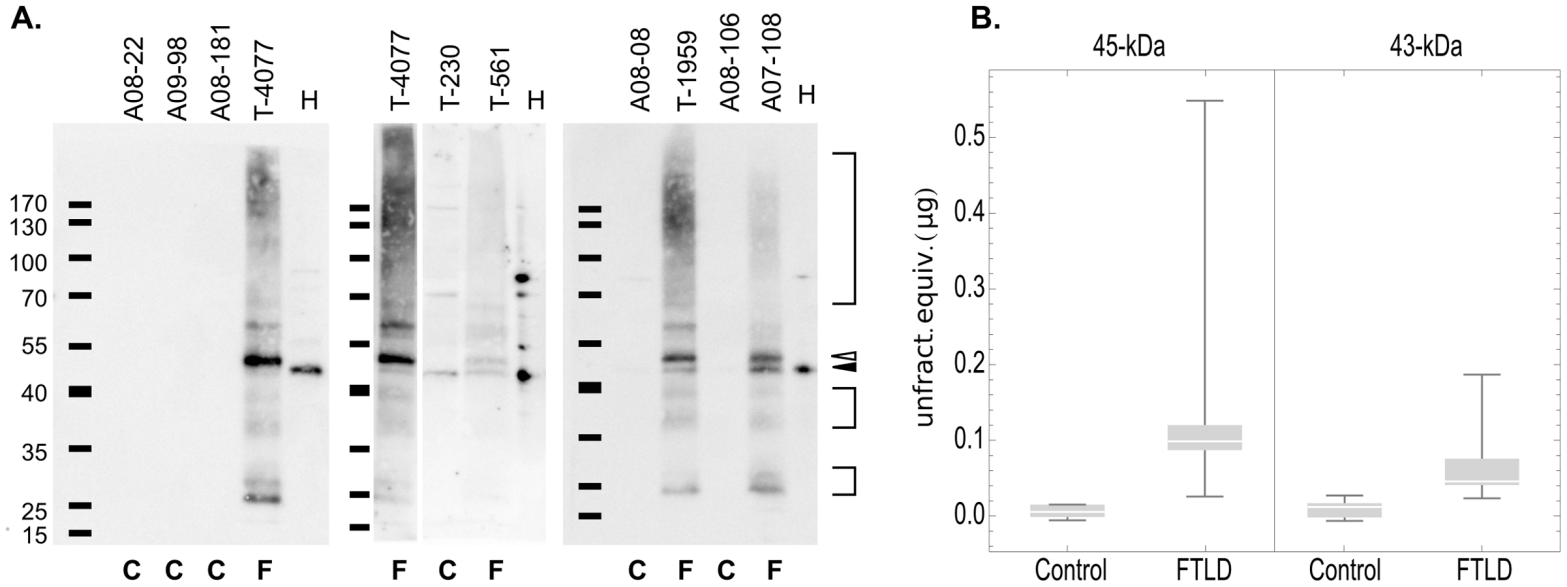
Aggregates include a 43-kDa form of TDP-43 without apparent covalent modifications. (A) SDS-PAGE gels loaded with concentrates of aggregate region from FTLD (F) and control (C) cases. A band of 43-kDa (filled arrowhead) is seen in FTLD brain homogenates, but is absent or barely visible in most controls. Several other forms of TDP-43 are seen only in FTLD cases: a pair bands near 25-kDa (lower bracket); a pair of broad bands near 37-kDa (middle bracket); a 45-kDa band (open arrowhead); an ∼60-kDa band, and a broad band of high molecular weight species (upper bracket). In each gel a lane loaded with 0.5 µg (total protein) of 10% brain homogenate (H) is included both to indicate the apparent molecular weight of the 43-kDa species of TDP-43 that predominates in brain, and as an indication of the relative quantity of TDP-43 isolated from the aggregate. The 3 panels represent the results of 3 independent preparations from brain tissue specimens. T-4077 is represented in the first and the second panel of the figure because it was part of two preparations. The positions of protein mass standards are indicated with horizontal bars. (B) Quantity of 43-kDa and 45-kDa bands in concentrates of the SDD-AGE aggregate region. Quantities were determined as described in the Methods and are expressed as equivalents, expressed as µg total protein loaded in the well, of the 43-kDa band from unfractionated control brain homogenate. The quantities of both the 45-kDa band (P = 0.0003, one-tailed T-test) and 43-kDa band (P = 0.021, one-tailed T-test) are significantly greater in FTLD than in control concentrates. The box-whisker plots depict the range (thin line), median (white line) and 25–75 percentile range (box) of values.

Having developed a method to efficiently concentrate proteins from excised regions of SDD-AGE gels, we proceeded to analyze the TDP-43 components of the aggregate region. SDS-PAGE immunoblots of concentrates from the aggregate region revealed several species of TDP-43 (Fig. 5A). All FTLD-TDP cases contained a broad band with greatest density at about 170 kDa, a pair of closely apposed bands of ∼60-kDa, bands of ∼45-kDa and ∼43-kDa, and two somewhat indistinct doublets centered at ∼37-kDa and ∼25-kDa. The 43-kDa band exhibits an identical migration to the 43-kDa species predominantly present in unfractionated brain homogenate. As expected, most of these TDP-43 bands are absent in concentrates of the aggregate region from control brains. A 43-kDa band is barely visible in some control cases, but in only one case does the density of the band approach that of FTLD-TDP cases. Quantitative analysis of these SDS-PAGE gels shows that the median amount of the 43-kDa band in FTLD-TDP aggregates is small, the equivalent of the 43-kDa band from 0.05 µg (total protein) of unfractionated brain homogenate. Given that approximately 22% of the concentrate from the aggregate region was loaded in each lane of the SDS-PAGE gel, that 400 µg (total protein) was loaded in each lane of the SDD-AGE gels, and that yield is approximately 77% from the excised region, then the 43-kDa band in aggregates represent approximately 1/1400 the total TDP-43 loaded on the SDD-AGE gel (Fig. 5B). However, the difference between the density of the 43-kDa bands between FTLD cases and controls is significant (p = 0.021, one-tailed T test). The entire preparation, from brain tissue through SDS-PAGE analysis, was performed at least twice on all cases, and essentially identical results seen each time.

Concentrates from the “monomer” region revealed a 43-kDa form of TDP-43 as the major species in both control and FTLD brain homogenates. Despite the apparently similar amounts of TDP-43 in the “aggregate and “monomer” regions in images of SDD-AGE immunoblots of FTLD brains, the amount to TDP-43 in the monomer region seemed to be much greater than in the aggregate region (data not shown). Precise quantitation was not possible because of the differing distributions of the TDP-43 species on PAGE immunoblots between the aggregate monomer regions.

**Figure 6:**
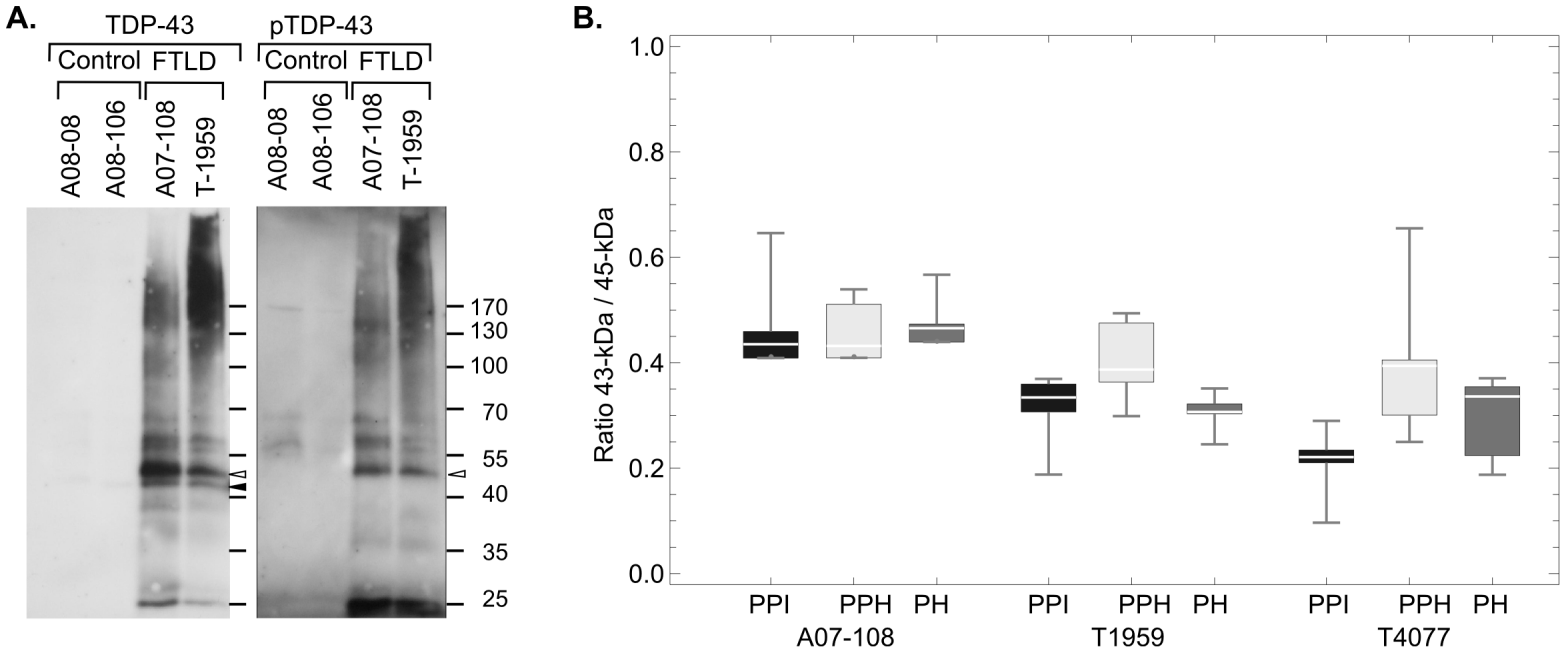
The 43-kDa TDP-43 band is not phosphorylated at S409/410 and not derived from 45-kDa band during processing. (A) Immunoblot prepared exactly as those in Fig. 5A**,** then stripped of bound antibody and re-probed with an antibody against pS409/410 TDP-43. Phosphorylated species correspond to most of the FTLD-specific bands seen in A**,** including the approximately 45 kDa band (open arrowhead), but the 43-kDa TDP-43 band (closed arrowhead) is not phosphorylated at the S 409/410 site. (B) Brain homogenates were subject to conditions designed to minimize or maximize phosphatase activity (see Methods), then proteins were concentrated from the aggregate region. The quantity of the 45-kDa and 43-kDa bands were determined as described in the methods, and the ratio of the 43-kDa to 45-kDa band calculated. The box-whisker plots depict the results (range, 25–75 percentile, median) of at least 5 measurements of the ratio. The figure depicts the ratio from homogenates of 3 FTLD cases either prepared with both protease and phosphatase inhibitor stored on ice (PPI), prepared with both inhibitors and heated (PPH), or prepared with only protease inhibitor and heated (PH). An increase in the ratio of the 43-kDa to 45-kDa species in the PH condition, relative to either the PPI or PPH condition would suggest that significant endogenous phosphatase activity present in sample processing might generate the 43-kDa species from the phosphorylated 45-kDa species. No consistent pattern is seen.

Immunoblots probed with a monoclonal antibody recognizing TPD-43 phosphorylated at serines 409 and 410 (pS409/410) reveal that most of the TDP-43 bands from FTLD-TDP aggregates contain phosphorylated TDP-43 species (Fig. 6A). However, the 43-kDa band present in the blots probed with an antiserum recognizing TDP-43 is absent when the same blot is re-probed with an antibody to pS409/410 TDP-43, indicating this 43-kDa species is not phosphorylated at these sites.

We considered whether endogenous phosphatase activity might have generated the 43-kDa TDP-43 species from a full-length phosphorylated species, such as that composing the 45-kDa band. To determine whether such activity was present during our aggregate preparation procedure, brain homogenates were prepared from frozen tissue under conditions designed to either minimize or enhance the activity of hypothetical phosphatases, then concentrates of the aggregate portion of these homogenates prepared. We predicted that if phosphatase activity were present in the homogenates, then the ratio of the 43-kDa species to the 45-kDa species would increase under the enhancing conditions. No significant effect was observed (Fig. 6B).

## Discussion

SDD-AGE separates aggregated TDP-43 species that are present in FTLD-TDP brain from forms of TDP-43 that are found in both FTLD-TDP brains and controls. SDD-AGE is thus a simple and rapid assay for aggregated TDP-43 that may prove useful in future studies of the nature and distribution of these pathological aggregates.

In SDD-AGE gels, the predominant lower molecular weight species in both controls and FTLD cases migrates with an apparent molecular weight of about 170 kDa, whereas is SDS-PAGE gels the normally predominant band migrates as a 43-kDa band. Since SDD-AGE only partially denatures proteins, migration rates would not be expected to be directly proportional to molecular mass. The apparent larger size of the predominant TDP-43 species in SDD-AGE gels may reflect slowed migration of this species in a partially unfolded state or interactions of the migrating TDP-43 peptide with other species.

Since the aggregates seen with SDD-AGE are present only in FTLD-TDP cases, all TDP-43 species migrating in these aggregates are likely to be disease-related. In contrast, the TDP-43 species isolated by the serial extraction method that is commonly used to enrich for FTLD-associated forms of TDP-43 also include a substantial amount of TDP-43 that is not specifically associated with disease [9], [17]. We sought to exploit the specificity of SDD-AGE to better define the TDP-43 species present in pathological aggregates.

TDP-43 aggregates are found in a region of SDD-AGE gels that contain only about 1% of the total protein in the lane. A number of proteins other than TDP-43 were isolated from this region in both FTLD cases and controls. Perhaps surprisingly, the TDP-43 bands revealed by immunoblots of protein from this region are not distinctly visible in silver-stained gels. This may reflect the low total amount of TDP-43 protein in this region, the diffuse nature of most TDP-43 bands or a lack of affinity of TDP-43 species for the silver stain. Whether other proteins from the aggregate region are also aggregated, whether they are in the same cellular compartment as aggregated TDP-43, and whether other proteins from this region are specifically found in FTLD is not known, but these are potentially fruitful areas for future studies.

The TDP-43 species extracted from the aggregates in SDD-AGE gels appear to include all the modified forms of TDP-43 forms that had been previously identified in the detergent-insoluble fraction from patients with FTLD-TDP [9], [18]. These include: (a) A broad band ranging from the well to at least 60-kDa with greatest density at about 200-kDa. At least some TDP-43 components of this band are phosphorylated, as demonstrated in our studies and others [18]. TDP-43 in this band is also ubiquitinated [9] (b) In our preparations a doublet is clearly seen in the 56-kDa-60-kDa range. These species are phosphorylated at S409/410. Although not commented on by previous authors, a similar doublet may be present in sarcosyl-insoluble preparations [9], [18]. (c) A 45-kDa species. This strongly reacts with anti-S409/410 phospho-TDP-43 antibody in our investigations and those of others [26]. This band was previously shown to convert to a 43-kDa species upon treatment with phosphatases [9]. It therefore appears to be phosphorylated full-length TDP-43. (d) An indistinct doublet in the 35-40-kDa range. Although these TDP-43 species were not emphasized in the original descriptions of the N-lauroylsarcosine-insoluble forms of TDP-43 associated with FTLD-TDP [9], they have been noted in subsequent studies [27] and may be C-terminal fragments generated by the caspase-mediated cleavage of TDP-43 at codon 89 [27], [28]. Our studies and those of others show it to be phosphorylated. (e) A pair of bands of about 25-kDa in mass. We and others find these to be phosphorylated. Other studies have indicated that these are C-terminal fragments of TDP-43 [9].

Our most significant finding, however, is that a 43-kDa species of TDP-43 is present in FTLD-associated TDP-43 aggregates. The aggregated 43-kDa species migrates in SDS-PAGE identically to the physiologically predominant TDP-43 species, and lacks obvious covalent modifications. Like the physiologically predominant species, it is not phosphorylated at the 409/410 sites. Known sites of TDP-43 phosphorylation in FTLD include serines at codons 379, 403, 404, 409 and 410 [18]. Our studies do not conclusively exclude phosphorylation at sites other than 409 and 410. However, we note that a phosphorylated 43-kDa species was not identified in the sarcosyl-insoluble fraction from FTLD or ALS tissue using antibodies against TDP-43 phosphorylation sites at S409, S410, S409/410, S403/404, or S379 [18]. Many types of covalent modifications of protein are known to occur, and some might not alter the migration of the protein in SDS-PAGE [29]. Without large amounts of purified protein it is not possible to exclude most such modifications. Nevertheless, our data suggest that the covalent structure of the 43-kDa species in FTLD-associated aggregates is identical to that of the normally predominant physiological form of brain TDP-43. The mass-yield of this species in our preparations is small, so it has not been possible to determine its structure by mass spectrometry.

This aggregated 43-kDa species is present at significantly higher levels in FTLD-TDP cases than in controls. To our knowledge, these studies represent the first demonstration of aggregated 43-kDa TDP-43 in diseased human brain. Immunohistochemical studies had indicated the presence of “full length” TDP-43 in intracellular inclusion in both FTLD-TDP and ALS, but it is not possible from such studies to know if the full-length TDP-43 had undergone phosphorylation or other covalent addition of a chemical group [20]. Some evidence that TDP-43 lacking covalent modifications can form aggregates exists in studies of cultured cells induced to undergo apoptosis, in which neither proteolytic processing nor phosphorylation are necessary for the formation of insoluble TDP-43 [27].

The forms of TDP-43 found in disease-associated aggregates may provide insight into the process of pathological aggregation in FTLD. To summarize, these species include at least 2 forms whose polypeptide length appears to be the same as the physiologically predominant 43-kDa species (a form not phosphorylated at S409/410 and a phosphorylated form); several truncated forms, at least some of which are phosphorylated; and forms with molecular weights greater than 45 kDa that are phosphorylated. Species other than the 43-kDa and 45-kDa forms may be ubiquitinated as well. (Whether all species migrating in bands that are immunoreactive with both phospho-TDP-43 and ubiquitin antibodies have these modifications is not determined).

From the observation that apparently full length TDP-43 is present in aggregates, one can conclude that truncation cannot be essential for aggregation because the processes leading to truncation are presumably irreversible. By similar reasoning, the presence TDP-43 appearing to lack phosphorylation suggests that phosphorylation is not essential for pathological aggregation. However, unlike truncation, phosphorylation is not necessarily irreversible. It is conceivable that endogenous phosphatase activity might generate 43-kDa TDP-43 from the 45-kDa species (or other phosphorylated full-length species) after aggregation had occurred. We found no significant endogenous phosphatase activity upon TDP-43 in our post-mortem tissue samples, but this does not exclude the possibility that such a process was active *in vivo* or in the interval between death and autopsy. Excepting this possibility, the presence of TDP-43 without apparent phosphorylation, truncation or ubiquitination in aggregates indicates that none of these covalent modifications is necessary for TDP-43 aggregation. This conclusion does not exclude that possibility that aggregation might be initiated by covalently modified forms, with full-length forms subsequently aggregating by another process triggered by truncated aggregates. Such processes might include impairment of quality control mechanisms that establish and maintain proper TDP-43 conformation or that clear aggregated TDP-43. In this regard, one study found that truncated forms of TDP-43 expressed in cultured cells recruit full length TDP-43 into aggregates [30].

Another possibility is that covalent modifications are not necessary to initiate and perpetuate TDP-43 aggregation. In the neurodegenerative conditions known as prion disease, the prion protein (PrP) converts to an aggregated form without covalent modification. Instead, pathological PrP aggregates seem to serve as a template for a conformational change in native PrP that drives further aggregation. Evidence exists for prion-like propagation of the aggregated proteins associated with several neurodegenerative diseases [31]. TDP-43 aggregation might be driven by a prion-like mechanism. The prion-like induction of TDP-43 aggregation by exogenously introduced fibrillary aggregates of TDP-43 was recently demonstrated in cultured cells [32]. In this scenario, covalent modifications such as phosphorylation, truncation and ubiquitination would occur after the 43-kDa species aggregates, perhaps as a cellular response to the presence of the aggregated protein.

The significance of apparently aggregated 43-kDa TDP-43 in one of our controls in an amount approaching that found in in FTLD cases is uncertain. A separate preparation from the same brain gave similar results (data not shown). Histopathologic examination of this brain did not reveal TDP-43 positive intracellular inclusions, but biochemical techniques may be more sensitive that immunohistochemistry for the detection of pathological protein aggregates [33]. The truncated, ubiquitinated and phosphorylated forms seen in FTLD-TDP cases were not observed. Whether the 43-kDa TDP-43 isolated from the high-molecular weight regions of SDD-AGE gels from controls and larger amounts found in FTLD-TDP cases are conformationally identical or in the same processing pathway remains to be determined.

## Materials and Methods

### Tissue homogenates

A waiver of approval was obtained from the Colorado Multiple Institutional Review Board because only autopsy tissue was used in these studies. Frozen blocks of human cerebral cortex from autopsy specimens were obtained from either the New York Brain Bank or the Neuropathology Division at the University of Colorado Denver. The clinical characteristics of all cases are summarized in Table 1. All FTLD cases were diagnosed immunohistochemically with anti-TDP-43 antiserum (New York Brain Bank samples) or anti-ubiquitin antibodies (University of Colorado samples). Since the University of Colorado FTLD cases were shown during the course of our studies to have abnormal TDP-43 aggregates, the term FTLD-TDP is used to refer to all FTLD cases described in this report. These tissues were harvested at times varying from 6 to 22 hours after death, then stored at −80°C until homogenates were prepared. Details of the method of collection of the New York Brain Bank specimens have been published [34]. For the University of Colorado specimens, cortical regions were identified by gross inspection. In all cases, an effort was made to include primarily cortical gray matter in the homogenate.

**Table 1:** Clinical information on sources of cerebral cortex samples used.

Case number	Age at death	Gender	Diagnosis
A07-108	43	M	FTLD-TDP
T-1959	79	M	FTLD-TDP
T-4077	66	M	FTLD-TDP
T-561	80	M	FTLD-TDP
T332	62	M	FTLD-TDP
A08-106	57	M	Encephalitis
A09-98	70	M	Ruptured abdominal aortic aneurysm
T-230	87	M	No neurodegenerative disease
A08-08	76	M	Primary systemic (AL) amyloidosis
A08-181	51	F	Depression
A08-22	51	M	ALS (without FTLD)
T-325	89	M	No neurodegenerative disease
A08-177	57	M	West Nile Virus Encephalitis

Case numbers of tissues from the University of Colorado, Denver Medical Center are prefixed with an “A”, those from the New York Brain Bank with a “T”. Diagnoses reflect the neuropathological diagnosis or, where there was no specific neuropathological diagnosis, the disease causing death, where known.

Brain tissue was homogenized in 9 volumes ice-cold 25 mM Tris, pH 7.4, with Complete inhibitor cocktail (Roche). Where indicated, a phosphatase inhibitor cocktail, PhosStop (Roche) was also added to the homogenization buffer. Tissue was suspended in buffer using a 5-mL Tenbroeck tissue grinder on ice, then clarified by centrifugation at 600 g for 30 seconds. Total protein concentration of homogenates was determined by the bicinchoninic acid assay (BCA, Thermo Fisher Scientific, Rockford, IL).

### Analytic SDD-AGE gels

This method is a modification of a technique used to demonstrate aggregated forms of yeast prion proteins [23], [24], [25]. Standard melting temperature agarose I (Amresco, Solon, OH) 1.5% (w/v) was melted in a buffer of 25 mM Tris (pH 8.3) and 192 mM glycine, then SDS was added to 0.1% (w/v). The hot solution was then poured into a mini-gel vertical casting assembly (Mighty Small, Hoefer, Inc., Holliston, MA) with 1.5-mm spacers to form a 10.5-cm long 8-cm wide gel. Prior to casting, the assembly was heated to 80°C in an oven to prevent premature gelling of the agarose solution. A 10-well comb was used. The gel was cooled at room temperature for 30–45 minutes. Electrophoresis was performed in the mini-gel electrophoresis apparatus per the manufacturers instructions for Laemmli SDS-PAGE except as described below. The buffer chambers were filled with a standard Laemmli running buffer (25 mM Tris pH 8.3, 192 mM glycine, 0.1% SDS). Brain tissue homogenates were mixed 1∶1 with a 2x stock of SDD-AGE loading buffer composed of 2% SDS, 80 mM Tris (pH 6.8), 25 mM dithiothreitol, 10% glycerol, and 0.04% bromphenol blue and briefly mixed by vortexing. This mixture was incubated at room temperature for 10 min, briefly agitated with a vortex mixer twice during this incubation, then loaded into the wells of the gel. Except where indicated, the samples were not boiled. Proteins smaller than approximately 75-kDa run faster than the bromphenol blue band in these gels, so migration was monitored with a pre-stained protein mass ladder (PageRuler, Fermentas, Glen Burnie, MD). Electrophoresis was carried out at 125 V with ice water cooling, until the 15-kDa marker reached the bottom of the gel. Masses of slowly-migrating bands were estimated by comparing the migration of bands in SDD-AGE immunoblots to bands in Coomassie-stained SDD-AGE gels run under identical conditions but loaded with chicken pectoralis muscle homogenate, which has prominent high-molecular-weight proteins [23], [35].

### Preparative SDD-AGE gels

We developed methods to isolate sufficient TDP-43 for further analysis from the high molecular weight regions of SDD-AGE gels (Fig. 3). A large format vertical slab electrophoresis apparatus (SE 600 Chroma, Hoefer, Holliston, MA) was used to prepare a 14 cm wide ×16 cm long ×0.15 cm thick gel. As with the analytic gels described above, standard melting temperature agarose I, 1.5% (w/v) was melted in a buffer of 25 mM Tris (pH 8.3) and 192 mM glycine, then sodium dodecyl sulfate (SDS) was added to 0.1% (w/v). The casting apparatus was assembled according to the manufacturers instructions, and was heated to 80° C prior to casting to prevent premature gelling of the agarose solution. The gel was cooled at room temperature for approximately 30 min. until the agarose solidified, and was then chilled at 5°C for 15 min.

The gel was next modified to permit separation of a large volume of homogenate by vertical electrophoresis. First, the comb was removed. To accomplish this without tearing the delicate gel, the clamps holding the glass plates and spacers were loosened and removed. The gel assembly was held parallel to the floor, and the top glass plate was carefully slid away from the comb, without introducing air between the plate and the underlying gel, to expose the comb. The comb was then removed. Large volume wells were made by removing the agarose barrier between 4 adjoining wells with a clean micropipette tip. The top plate was then carefully slid back into position and the clamps were replace. In order to prevent the gel from sliding out of the bottom of the assembly during vertical electrophoresis, a 1 cm wide retention band was poured at the bottom of the gel: First, the assembly was held in an upright position until the gel gradually slid out from between the glass plates, exposing a 1 cm wide band. This was excised with a clean razor blade. The assembly was then inverted until the gel returned to its original position. The assembly was then placed horizontally on the bench top. Three strips of blotting paper, 1 cm ×14 cm were then insert into the 1 cm gap at the bottom of the gel. A 30% polyacrylamide solution (30% acrylamide (w/v), 0.6% bis-acrylamide (w/v), 20 mM Tris (pH 7.4) and 200 mM glycine, 0.1% SDS (w/v), 5 µL/mL 10% ammonium persulfate, 0.5 µL/mL TEMED) was then carefully pipetted onto the paper and allowed to fill the 1 cm gap by capillary action.

When the polyacrylamide retention band polymerized, the gel was placed in the apparatus according to the manufacturers instructions. The buffer chambers were filled with a standard Laemmli running buffer. Samples were prepared by mixing 400 μg (total protein) of homogenate, typically about 100 μL, with an equal volume of 2× SDD-AGE loading buffer and incubated at room temperature for 10 min with 3 brief agitations using a vortex mixer. The electrophoresis was performed at constant 125 V with ice water cooling until a pre-stained 15-kDA marker (Pageruler, Fermentas) reached the top of the polyacrylamide strip. Regions of interest were excised from the gel with a clean razor blade. Up to 4 samples can be processed in parallel with this method using a single 2-gel apparatus. At least 1 control and 1 FTLD-TPD case were included in each run of the method.

### Concentration of proteins from SDD-AGE gel regions

We modified the “three-layer sandwich gel electrophoresis” method [36] to efficiently concentrate proteins from the excised regions of SDD-AGE gels. Borosilicate glass tubes (Electro-eluter Model 422, Biorad), 6 cm long ×1 cm internal diameter, were prepared as follows. A 40% acrylamide solution in a buffer of pH 11 was prepared as described [36]. One mL of this solution was pipetted into upright glass tubes that had been sealed by pressing the tube into a 4-strip thickness of warm paraffin film on the bench top. The solution was overlayed with 200 µL water-saturated butanol. When the acrylamide solution had polymerized, the silicone adaptors supplied with the electoeluter were affixed to the glass tube to help retain the polyacrylamide plug. The tubes were rinsed with ultrapure (18.2 MΩ Milli-Q) water.

Meanwhile, excised portions of large format SDD-AGE gels were weighed. SDS was added to 1% and dithiothreitol to 25 mM. Samples were boiled for 5 minutes to melt the agarose and to denature protein complexes contained within it. Ultrapure water was added to a final volume of 4 mL, and the samples were briefly boiled again. The hot agarose solution was then pipetted into the glass tube over the sealing layer of polyacrylamide. After gelling, the agarose was capped with 100 µL of a melted solution of 0.01% (w/v) bromphenol blue in 1% agarose (w/v).

The tubes were loaded onto the Electro-eluter apparatus according to the manufacturers instructions, with the polyacrylamide sealing layer down. Electrophoresis was performed in Laemmli buffer at 15 V for 30 min, reduced to 5 V for 16 hr., then increased stepwise to 140 V over 5 hours. Under these conditions, bromphenol blue migrated to form a thin band just above the polyacrylamide sealing layer. The agarose column was extruded from the tube by running a 30-gauge needle of a 1 mL tuberculin syringe around the perimeter of the polyacrylamide plug to loosen its adherence to the glass tube, then pushing on the polyacrylamide plug with the thumb rest of the syringe. An approximately 1.5 mm wide slice of agarose encompassing the bromphenol blue band, and where appropriate, sequentially higher slices were excised with a clean razor blade for further analysis.

### Polyacrylamide gel electrophoresis and immunoblotting

SDS-PAGE was performed using standard Laemmli technique with precast tris-glycine gels (Invitrogen) [37]. In the case of samples concentrated in agarose columns, the approximately 120 µL of agarose containing the concentrated proteins was weighed then mixed 3∶1 with 4x Laemmli buffer and boiled. This hot mixture remained liquefied long enough to load into the wells of SDD-PAGE gels by underlaying in the usual manner.

For immunoblotting of both SDS-PAGE and SDD-AGE gels, proteins were transferred to polyvinylidene fluoride (PVDF) membrane using a mini-tank (TE 22, Hoefer, Holliston, MA) using Towbin buffer per the manufacturers instructions [38]. The analytic agarose gels were handled just as polyacrylamide gels. Blots were probed with polyclonal antiserum (10782-2-AP, Proteintech Group, Chicago, IL) or murine monoclonal antibody 2E2-D3 (Abnova, Taipei City, Taiwan), both recognizing human TDP-43; and the appropriate horseradish peroxidase (HRP)–conjugated secondary antibody. These were developed using the ECL Plus kit (Invitrogen, Carlsbad, CA).

For analysis of phospho-TDP-43 bands, blots were first probed and imaged using polyclonal antiserum 10782-2-AP as above. The blot was then stripped of antibody using a commercial stripping buffer (Restore, Thermo Scientific) according to the manufacturers instructions. The blot was then probed with anti-phospho-TDP-43 (Ser409/Ser410), clone 1D3 (Millipore, Billerica, MA) and anti-rat IgG-HRP conjugate secondary antibody.

SDS-PAGE gels for silver staining were run with unstained protein mass standards (Benchmark, Invitrogen). Silver staining was done using a SilverXpress kit (Invitrogen) per the manufacturers instructions. Coomassie staining was done using Brilliant Blue G (Sigma-Aldrich, St. Louis, MO) following the protocol supplied by the manufacturer.

### Studies of phosphatase activity

Two brain homogenates were prepared from each of 3 FTLD cases. Tissue was homogenized using a 1 mL Tenbroeck tissue grinder on ice, in 9 volumes ice-cold 25 mM Tris, pH 7.4 with either Complete protease inhibitor cocktail or both Complete protease inhibitor and PhosStop phosphatase inhibitor cocktail. Samples were stored on ice while protein concentrations were determined, then 800 µg (total protein) aliquots of both protease inhibitor only or protease inhibitor and phosphatase inhibitor treated homogenates either stored on ice or incubated at 37°C for 1 hour. The samples were then subject to large volume SDD-AGE followed by concentration of the aggregate region, then separated by SDS-PAGE as described above. Immunoblots were made using polyclonal anti-TDP-43 antiserum. The 43-kDa and 45-kDa bands were quantified as described below, and ratio of the 43-kDa band to the 45-kDa band was determined.

### Denaturation studies

Urea was prepared in 0 M, 2 M, 6 M and 8 M concentrations in 50 mM Tris pH 6.8, and 15 µL of the urea solution was mixed with 5 µL of a 10% brain homogenate, prepared as described above, from an FTLD case. The mixtures were vortexed briefly and incubated for 60 minutes at room temperature. The samples were then processed for SDD-AGE and immunoblotting. To assess the effect of boiling in the presence of SDS and DTT, 5 µL of 10% brain homogenate was mixed with 5 µL of SDD-AGE loading buffer. One sample was held at room temperature for 10 minutes (per SDD-AGE protocol described above) while the other was heated to 100°C for 5 minutes. The samples were then processed for SDD-AGE and immunoblotting.

### Quantitative and statistical analysis of immunoblots and silver stained gels

All immunoblots were either directly imaged in a digital format, using a Kodak Image Station 4000 (Carestream, Rochester, NY) cooled-CCD digital camera, or converted to digital images from photographic film (Hyperfilm, GE Healthcare) by taking digital photographs of film images on a trans-illuminating stage (Kodak EDAS 290, Carestream, Rochester, NY). Digital images of Coomassie and silver stained gels were obtained by photographing them on a trans-illuminating stage.

For quantitative analysis, digital images were imported into Mathematica software [39] notebooks. The relative quantity of TDP-43 on western immunoblots was determined from digital images using the following procedure: Dilutions of a standard were subjected to SDS-PAGE or SDD-AGE either on the same blot or in tandem with the experimental blot. Tandem blots were incubated with antibody in the same container and exposed simultaneously on the same piece of photographic film or in the same frame of view of the CCD camera. The standards were chosen so that the TDP-43 band pattern of the standard was similar to the bands of interest. Thus, dilutions of FTLD-TDP43 case A07-108 were used for relative quantitation of TDP-43 on SDD-AGE blots. The density of the upper band (upper bracket in Figure 1A) in dilutions of the standard was used to determine relative quantities of the upper band in samples, while the lower band (lower bracket, figure 1A) was used for the lower band in samples. For relative quantification of the 43-kDa and 45-kDa bands in SDS-PAGE immunoblots, dilutions of unprocessed control brain homogenate of known total protein concentration were used, and both 45-kDa and 43-kDa bands of FTLD cases were compared to the 43-kDa band from the standard. Quantities were thus determined as equivalents of the 43-kDa band in given quantity (total protein) of control brain homogenate.

Plots of apparent density versus dilution or quantity of the standard were prepared for each band of interest. Either 1^st^ (linear) or 3^rd^ order polynomial curves were fit to the standard density dilution plots using the Mathematica “FindFit” function. The order of the fitted curve was chosen empirically to minimize residuals. In most cases photographic film showed a sigmoidal response best fit to a 3^rd^ order curve, while the directly obtained CCD camera images showed a linear response. The equations for these fitted curves were applied to the apparent densities of the cognate bands in the experimental blot, so that quantities were determined relative to the dilution of the standard. This relative quantitation was used in comparisons between samples in different experiments. Absolute TDP-43 quantities were not determined. In measuring densities of bands, median pixel values were used instead of mean values because this method was less sensitive to irregularities such as specks and blank spots in the bands images. Particularly when measuring closely apposed bands, such as the 43-kDa and 45-kDa species, the measured density depended on the precise positioning of the region of interest. To minimize error and variance due to positioning the region of interest, each ROI median density was measured 5 times and the mean value determined.

To determine yields from the concentration process that we developed, portions of an SDD-AGE gel region concentrate were loaded onto a PAGE gel with serial dilutions of a standard. In the case of aggregate region concentrates, whole brain homogenate of known protein concentration was the standard. In the study of the efficiency of pre-stained marker recovery from the gel, the same lot of pre-stained markers was the standard. The pre-stained marker gel was imaged unstained, otherwise the PAGE gel was silver stained. A plot of standard dilutions versus mean whole lane density was made in Mathematica, and the concentration of the aggregate region concentrates determined by fitting to this curve, using the method described above for determining individual band densities. Yields were then calculated from these concentrations.

Statistical tests were performed using Mathematica software. Comparison of band densities between FTLD cases and controls were made using the Mathematica “TTest” function, which performs a Student T-test on univariate data such as in this study. Because it was not clear that the density values would be distributed normally, a non-parametric test was also applied. In all cases the T-test yielded the highest, i.e. least significant therefore most conservative, P value and this was reported.
